# Efficacy of acupuncture and rehabilitation therapy on brain function activation area and neurological function in ischemic stroke: A systematic review and meta-analysis

**DOI:** 10.1371/journal.pone.0298547

**Published:** 2024-02-23

**Authors:** Tao Zhu, Yihao Zhou, Anhong Dai, Song Li, Li Zhou, Xiahui Zhang, Wei Zhang, Jing Shi

**Affiliations:** 1 Yunnan University of Chinese Medicine, Kunming, China; 2 The First Affiliated Hospital of Yunnan University of Chinese Medicine, Yunnan Provincial Hospital of Traditional Chinese Medicine, Kunming, China; 3 Heilongjiang University of Chinese Medicine, Harbin, China; 4 Yan’ an Hospital Affiliated to Kunming Medical University, Kunming, China; The Second Affiliated Hospital of Shandong First Medical University, CHINA

## Abstract

**Background:**

The probability of motor deficits after stroke is relatively high. At the same time many studies have reported that acupuncture and rehabilitation therapy have a significant effect on the treatment of stroke.

**Objective:**

This systematic review and meta-analysis aimed to evaluate the clinical value of acupuncture and rehabilitation therapy on brain eloquent areas and neurological function in ischemic stroke.

**Methods:**

Seven databases were electronically searched to screen randomized controlled trials (RCTs) of different intervention methods (acupuncture, rehabilitation) in the treatment of ischemic stroke. The search time is from January 1, 2000 to April 20, 2023, and the search languages are limited to Chinese and English. Two researchers independently screened literature and extracted data. The methodological quality of the studies was assessed using the Cochrane Handbook for Systematic Reviews of Interventions.

**Results:**

A total of 17 randomized controlled studies were included, including 699 patients, with a maximum sample size of 144 cases and a minimum sample size of 11 cases. Among them, 3 studies reported the brain function in SM1 area. The effective rate of the experimental group was higher than that of the control group [relative risk (OR) = 3.24, 95%CI: 1.49 to 7.05, *P* < 0.05]. The FMA score of patients in the experimental group was higher than that in the control group [mean difference (MD) = 4.79, 95% CI: 3.86 to 5.71, *P* < 0.00001]. The NIHSS score of patients in the experimental group was lower than that in the control group [mean difference (MD) = -4.12, 95% CI: -6.99 to -1.26, *P* < 0.05].None of studies reported adverse events.

**Conclusions:**

Acupuncture rehabilitation for ischemic stroke can activate corresponding brain functional areas and improve neurological deficits. The therapeutic effect of acupuncture rehabilitation treatment is better than that of basic western medicine treatment, and it is more effective in improving neurological deficits. At the same time, clinical research needs to use high-quality randomized double-blind controlled trials with more detailed and larger sample designs, long-term efficacy evaluation and evidence-based research methods.

## 1. Introduction

Stroke, including ischemic stroke and hemorrhagic stroke, is a leading cause of sudden death or disability. Ischemic stroke, also known as cerebral infarction, caused by blockage or narrowing in the arteries supplying blood and oxygen to the brain, is a common and recurring disease that threatens human health [1,2]. It has the characteristics of high morbidity, high disability rate, and high mortality rate, and it also has a higher recurrence rate than healthy people. In the early stage of ischemic stroke, cerebral ischemia directly reduces the supply of glucose, oxygen and other nutrients, leading to energy deficiency or consumption of nerve cells, and finally cell edema, necrosis, demyelination, and axonal demyelination other changes [3]. With the development of modern economy and social progress, the lifestyle of residents has been changed, and the risk factors of cerebrovascular diseases have been widely exposed. Ischemic stroke has become a high-incidence disease with the first disability rate and the second fatality rate in China [4]. Motor dysfunction is the most common symptom of ischemic stroke, and more than 70% of stroke survivors suffer from different degrees of motor dysfunction [5]. Approximately 85% of patients with stroke have upper limb dysfunction, and more than 60% of them have persistent hand dysfunction and are unable to live independently after treatment [6]. Among movement disorders, poststroke dyskinesias can also be divided into hyperkinesias, characterized by excessive, abnormal involuntary movements, and hypokinesias, characterized by lack or slowness of movement (bradykinesia) [7].When a disease occurs on one side of the brain due to ischemia and hypoxia, the arousal activity of the brain on the affected side decreases, while the activity of the cerebral cortex on the healthy side increases, and the high inhibition of the healthy side on the affected side limits the arousal activity of the cortex on the affected side, resulting in abnormalities in the body tissues controlled by it. When the brain tissue of the precentral gyrus is lesioned, limb movement dysfunction will occur [8].

Ischemic stroke can cause local hypoxia of brain tissue, which can lead to brain damage. Modern medical treatment methods include anticoagulant therapy, nerve cell protection, establishment of collateral circulation, strengthening of nutritional nerves, and certain gene therapy. In anticoagulant therapy, the use of heparin anticoagulant drugs can inhibit the spread of embolism, avoid embolism, accelerate thrombolytic and vessel unblocking, and at the same time promote the establishment of collateral circulation, slow down progressive neurological damage, and effectively prevent disease recurrence [9]. Due to various factors such as the timing of intervention treatment and treatment methods, the degree of recovery of many patients is also different, which will cause necrosis of part of the brain tissue and damage to nerve function to a large extent, leaving sequelae and reducing the patient’s quality of life [10].

In recent years, more and more studies have shown that acupuncture have significant curative effects in treating stroke, which can improve patients’ neurological and limb functions, relieve sequel symptoms, and improve patients’ quality of life [11]. Acupuncture can promote local blood circulation and nutrient metabolism, increase blood supply, and accelerate the repair of neuronal tissue. The therapeutic effect on ischemic stroke includes the release of hematoma substances, blood-brain barrier, inflammatory response, immune response, and local aerodynamics. Modern medical research [12–15] shows that acupuncture can improve the blood supply to some brain regions of hemiplegia patients, strengthen the recovery of nerve function, reduce the expression level of inflammatory factors, balance muscle tension, and relieve muscle spasm. The traditional concept of treating brain diseases in the field of traditional Chinese medicine is increasingly combined with modern neurosurgical technology to scientifically and objectively explain the essence of brain cell damage. In the early stage of ischemic stroke, early intervention and early treatment are needed to alleviate the harm caused by brain tissue damage, and rehabilitation therapy has become an indispensable treatment method. There are many rehabilitation treatment methods now, and Exercise rehabilitation is extremely important in movement dysfunction. The purpose of motor rehabilitation is to establish connections between as many neurons as possible in a short period of time, induce the generation of new motor nerve pathways, achieve further consolidation, and restore the innervation and motor control of cranial nerve function [16]. Rehabilitation, which can promote spontaneous neural recovery and experience-dependent plasticity, is the main approach used to promote functional recovery and independence in stroke patients [17]. Fine motor dysfunction of the upper limbs after stroke, though a vital challenge, is a necessity of post stroke treatment in order to reduce the stroke disability rate. Currently, the most commonly used rehabilitation therapies include physical factor therapy, exercise and occupational therapy, compensatory training, motor imagery therapy, contralateral C7 nerve root transfer, acupuncture, and massage [18]. Acupuncture and rehabilitation treatment of ischemic stroke have been widely recognized by clinical experts and are better diagnostic and treatment methods in the treatment plan. Therefore, when studying ischemic stroke, we must focus on these two treatment methods and conduct in-depth research. To explore its treatment mechanism, to further find effective evidence-based evidence for acupuncture and rehabilitation in the treatment of ischemic stroke, and to use more precise targeted treatments to reduce the harm caused by ischemic stroke.

Modern imaging detection technology is becoming more and more sophisticated. The effect of acupuncture on brain function has become the focus of brain science research. Resting-state functional magnetic resonance imaging (rs-fMRI), one of the methods for measuring neurological performance in humans, is often used to assess participants’ resting-state networks [19,20]. In order to better understand the characteristics of activated brain regions in acupuncture treatment of ischemic stroke, more and more acupuncture studies are applying rs-fMRI to clinical trials. However, most clinical trials have small sample sizes, which may also be due to differences in experimental design between studies, and no consensus has been reached [21]. For example, studies [22] have shown that acupuncture produces more pronounced changes in cortical activity in sensory, emotional, and motor regions, including the frontal lobe, middle temporal gyrus, cerebellum, and lobes. Liu [23] reported that acupuncture at acupoints in the scalp acupuncture group can specifically enhance the local functional activities of the sensory, language, and motor coordination-related brain regions in patients with acute infarction in the dominant cerebral hemisphere, and at the same time strengthen the connectivity of the bilateral frontal lobe motor-related brain regions.

The current research has achieved a lot of results in acupuncture and rehabilitation in the treatment of specific brain regions of ischemic stroke, but most of them lack high-level and strong evidence due to insufficient sample size and other research design issues. Therefore, in order to scientifically evaluate the specificity of acupuncture and rehabilitation in activating brain functional areas and improving neurological function in the treatment of ischemic stroke, further analysis and integration are carried out to understand the specific effects of acupuncture on stimulating brain areas, and to clarify the effects of acupuncture and rehabilitation.

## 2. Methods

### 2.1 Protocol and registration

We conducted the meta-analysis in line with the Cochrane Handbook for Systematic Reviews of Interventions. This study is registered in the PROSPERO International Register of Prospective Systematic Reviews (Registration Number: CRD42023424015). Our study was strictly implemented adhere to the Preferred Reporting Items for Systematic Reviews and Meta-Analyses (PRISMA) reporting guidelines [24]. The PRISMA Checklist is shown in S1 Appendix.

### 2.2 Literature search

We searched the literature on patients with ischemic stroke from seven databases to compare the activation of acupuncture and rehabilitation on the activation of brain function activation areas. Three investigators (ZT, ZL, LS) searched seven databases from January 1, 2000 to April 20, 2023, and restricted the language to bilingual Chinese and English. The seven databases included three English language databases (Cochrane Library, Embase, and PubMed) and four Chinese language databases (China National Knowledge Infrastructure (CNKI) Database, Chinese Science and Technology Periodical (VIP) Database, Wan Fang Database, and Chinese Biological Medicine Database (CBM)). Taking "acupuncture" as an example, we used the following English search terms: (Cerebral infarction OR Cerebral infarction OR Stroke OR Ischemic stroke OR Cerebrovascular accident) and (Acupuncture OR Acupuncture OR Body acupuncture OR Head needle OR Electroacupuncture OR Warm acupuncture OR Acupuncture) and (fMRI OR MRI OR Functional magnetic resonance OR Resting magnetic resonance OR Brain function) and (RCT OR randomized controlled trial OR controlled clinical trial OR randomized OR clinical trial OR randomly OR RCT OR trial). The search strategy of each database is based on its own characteristics. The specific search strategy can be found in the S2 Appendix.

### 2.3 Types of studies

We selected studies of clinical randomized controlled trials, and explore the effect of acupuncture and rehabilitation therapy on the distribution of brain function activation area and nerve function in patients with ischemic stroke.

### 2.4 Inclusion/Exclusion criteria

The included studies were unrestricted for age, sex, ethnicity, race, economic status, and whether patients were inpatient or outpatient. Studies were included based on the following criteria: (1) Study subjects met the diagnostic criteria of ischemic stroke patients; (2) The interventions include acupuncture or rehabilitation; (3) The control group was sham acupuncture or basic treatment or other treatment methods of blank control; (4) Study outcome contains description of brain functional activation areas; (5) Published randomized controlled trials (RCT), the study protocol is reasonably designed, studies with full data recording and analysis;

Exclusion criteria include: (1) The sample size of each experimental group is less than 5; (2) The diagnosis of the disease is the diagnosis of post-stroke symptoms; (3) If different papers use the same or similar datasets, only the largest sample is included; (4) Animal studies, dissertations and conference papers, case reports, full text not available or no data available, and review articles were excluded;

### 2.5 Literature screening and data extraction

First, two researchers (LZ, SL) independently screened the retrieved literature according to the inclusion and exclusion criteria, and then any controversial literature was then assessed and discussed by a third reviewer (TZ) for cross-checking to reach a consensus. Data were extracted from the included studies into a standard form with respect to name of the first author, publishing year, participant status, sample size, interventions, brain function activation zones, and other evaluation indicators, and so on. Missing or unclear data were obtained by contacting the original authors by mail or telephone.

### 2.6 Quality evaluation

Since all included studies were randomized controlled trials, the quality of the included literature was evaluated by two reviewers (TZ; SL) independently using Version 2 of the Cochrane risk-of-bias assessment tool (ROB 2). The assessment tool focused on the randomization process, deviations from the intended interventions, missing outcome data, measurement of the outcome, selection of the reported result, and other bias. Each entry was judged to be: low risk, high risk, or some concerns. Any discrepancies regarding the assessment were resolved through discussion with a third investigator.

### 2.7 Data analysis

RevMan 5.3 software (The Cochrane Collaboration, Copenhagen, Denmark) was used to conduct meta-analysis on the data, and the heterogeneity between the studies was determined by χ^2^ test combined with *I*^2^ quantitative analysis. If *P* > 0.1 and *I*^2^ < 50%, it was considered that there was no obvious heterogeneity among the included studies, and the fixed effects model was used for meta-analysis; If *P* < 0.1 and *I*^2^ > 50%, it was considered that there was obvious heterogeneity among the included studies.

## 3. Results

### 3.1 Literature

**Fig 1:**
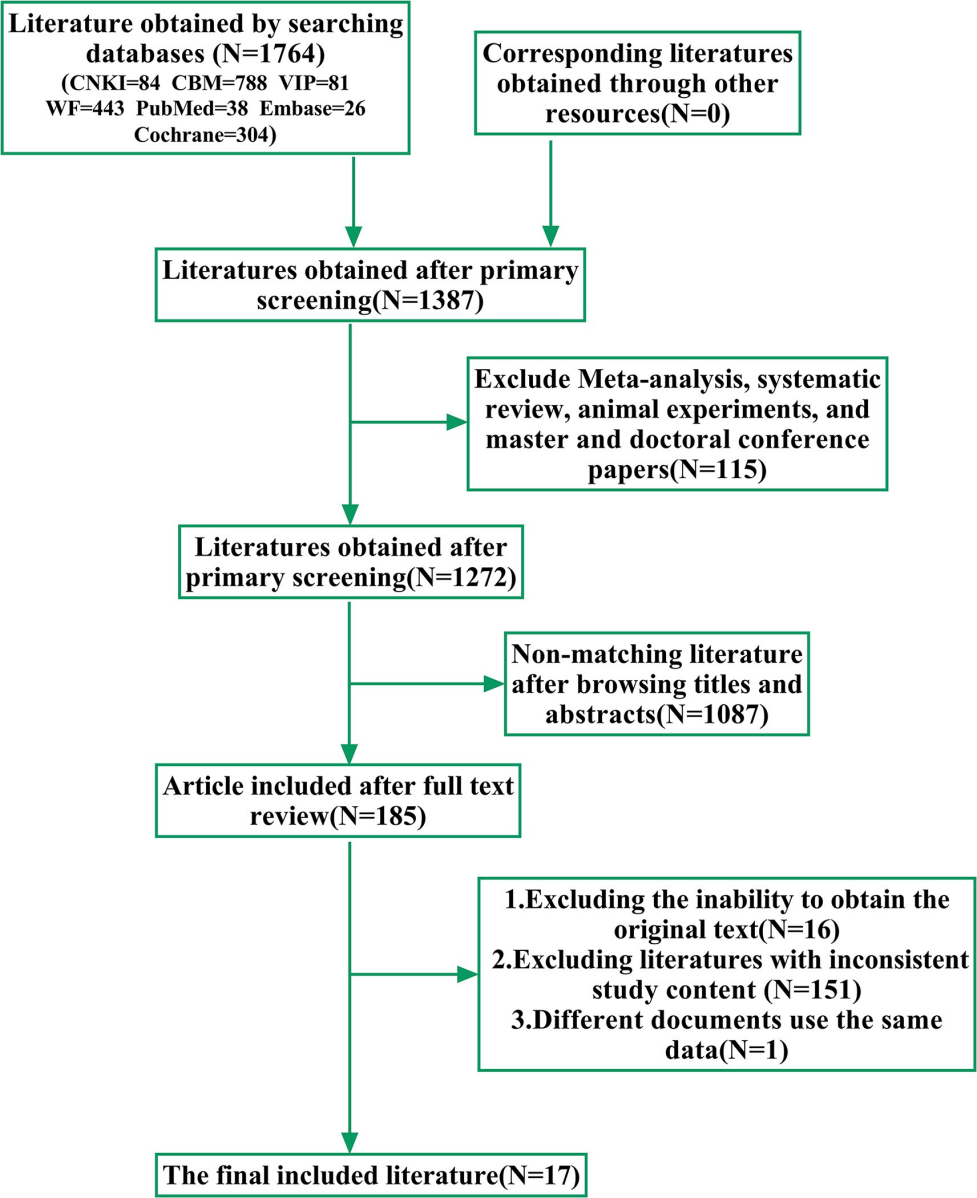
Literature screening process and results.

According to the literature search results, 1764 documents were initially obtained. 377 duplicate articles were excluded, 115 systematic reviews, animal experiments and other articles were excluded, and 1087 articles were excluded after screening titles and abstracted. After gradual screening, 17 studies were included [25–41], The literature screening process is shown in Fig 1.

### 3.2 Basic features of trials

All included studies were randomized controlled trials, including 699 patients. Among the included studies, the largest sample size was 144 cases, the smallest sample size was 11 cases, and the total number of patients in the experimental group was 351 cases, and the control group was 348 cases. All studies included patients with ischemic stroke, and the demographic baseline characteristics were balanced between the two groups. Both groups received usual care, and on this basis 10 studies reported adding acupuncture to patients in the treatment group and 7 studies adding rehabilitation to the treatment group. All studies reported fMRI results that illustrate brain function activation. Some studies also used the Fugl-Meyer Limb Motor Function Scale score (FMA), Barthel Index (BI), Modifified Barthel Index (MBI), the National Institutes of Health Stroke Scale (NIHSS), Magnetic Resonance Spectroscopy (MRS), FAC assessment, nervous functional defificiency scale (NDS), TCM Syndrome Scale, STEF Scores, BBT rating scale, the activity of daily living index scale (ADL). The detailed information and characteristics of the included studies are shown in Table 1.

**Table 1:** Basic information on the relevant literature included in the study.

Study	Year	Gender		Age	Random method	Cases		Intervention		Brain function activation	Outcome measures
		Male	Female			TG	CG	Test group	Control group		
**Li**	**2022**	**28**	**32**	**65±3**	**Random number table method**	**30**	**30**	**Acupuncture**	**Basic treatment**	**Frontal lobe**	**NIHSS、 FMA-UE、 fMRI**
**Zhao**	**2017**	**/**	**/**	**/**	**Random number table method**	**20**	**20**	**Acupuncture combined with traditional Chinese medicine treatment**	**Basic treatment**	**Parietal lobe, frontal lobe, cerebellum**	**NIHSS、 Effective RATE、 fMRI**
**Li**	**2015**	**/**	**/**	**/**	**Random by lottery**	**30**	**30**	**Acupuncture**	**Basic treatment**	**Temporal, frontal, insular, occipital, parietal lobes**	**fMRI**
**Gu**	**2013**	**17**	**23**	**55±8**	**Random number table method**	**20**	**20**	**Electroacupuncture**	**Basic treatment**	**Parietal, temporal, occipital, basal ganglia**	**FMA、 fMRI**
**Cai**	**2012**	**20**	**16**	**67.7±10.6**	**Not mentioned**	**18**	**18**	**Electroacupuncture plus finger movement therapy**	**Basic treatment**	**Frontal lobe, parietal lobe**	**fMRI**
**Wu**	**2017**	**/**	**/**	**/**	**Computer random**	**11**	**10**	**Acupuncture**	**Basic treatment**	**Frontal lobe, parietal lobe, temporal lobe, cerebellum**	**NDS、 FMA、 MBI、 fMRI**
**Liu**	**2021**	**22**	**8**	**59±11**	**Random number table method**	**15**	**15**	**Scalp Acupuncture**	**Basic treatment**	**frontal, parietal, temporal lobes**	**NIHSS、 FMA、 fMRI**
**Liu**	**2020**	**9**	**4**	**54±7**	**Random number table method**	**6**	**7**	**Scalp Acupuncture**	**Basic treatment**	**Parietal lobe, occipital lobe, cerebellum**	**NIHSS、 fMRI**
**Zhang**	**2012**	**/**	**/**	**/**	**Random by lottery**	**10**	**10**	**Acupuncture**	**Basic treatment**	**Temporal lobe, frontal lobe, insular lobe, occipital lobe, parietal lobe, basal ganglia, cerebellum, pons**	**fMRI**
**Ye**	**2018**	**46**	**38**	**56±8**	**Random number table method**	**42**	**42**	**Shengyang Tongdu Acupuncture Method**	**Basic treatment**	**Insula, parietal, frontal lobe**	**TCM SIC、 BI、 FMA、 fMRI**
**Zhang**	**2020**	**8**	**3**	**50±5**	**Random number table method**	**6**	**5**	**Motor imagery training**	**Basic treatment**	**Parietal lobe, frontal lobe**	**FMA、 STEF、 MBI、 fMRI**
**Gu**	**2020**	**14**	**2**	**60±10**	**Random number table method**	**8**	**8**	**Graded motor imagery**	**Basic treatment**	**Parietal lobe, frontal lobe**	**FMA、 BBT、 fMRI**
**Li**	**2020**	**19**	**23**	**62±7**	**Random number table method**	**21**	**21**	**Action Observation Therapy with Mirror Neurons**	**Basic treatment**	**Parietal lobe, frontal lobe**	**NIHSS、 FMA、 BI、 fMRI**
**Zhang**	**2019**	**72**	**72**	**59±4**	**Random number table method**	**72**	**72**	**Movement Observation Therapy Rehabilitation**	**Basic treatment**	**Frontal lobe**	**BI、 fMRI**
**Shen**	**2017**	**25**	**15**	**46±14**	**Random number table method**	**20**	**20**	**Movement observation therapy Rehabilitation**	**Basic treatment**	**Parietal lobe, frontal lobe**	**FMA、 BI、 fMRI**
**Gong**	**2017**	**15**	**13**	**43±9**	**Random number table method**	**14**	**14**	**Feedback functional electrical stimulation**	**Basic treatment**	**Parietal lobe, frontal lobe, cerebellum**	**FMA、 fMRI**
**Xing**	**2013**	**/**	**/**	**47±11**	**Random number table method**	**8**	**6**	**Feedback Functional Electrical Stimulation Therapy**	**Basic treatment**	**Frontal lobe**	**FMA、 fMRI**

Notice: FMA is the Fugl-Meyer Limb Motor Function Scale score; BI is the Barthel Index Rating Scale for Activities of Daily Living; The MBI is an improved Barthel index scale for assessing the ability to perform daily living; NIHSS is an NIHSS score on the National Institutes of Health Stroke Scale; MRS is a magnetic resonance spectroscopy detection technology; FAC is a functional walking grading scale that assesses a patient’s walking ability; NDS stands for Neurological Deficit Scale; TCM Syndrome Scale is an integral table for assessing TCM symptoms; STEF Scores the Short Upper Body Skills Examination for Hand Motor Coordination and Strength; BBT is a rating scale for testing general skill of the hand; ADL is the activity of daily living index scale; fMRI is functional magnetic resonance examination.

In the included studies, the most activated brain functional areas were the frontal lobe; parietal lobe, and temporal lobe, 15 studies activated the frontal lobe, accounting for 88.24% of the included studies. There are 14 studies that activated the parietal lobe area, accounting for 82.35% of the included studies; 5 studies activated 29.41% of the temporal lobe area. In the included studies, the lowest activation in the pontine region, Zhang [26] used acupuncture to activate the function of pontine brain area, accounting for 5.88%. The activation of brain function is shown in Table 2.

**Table 2:** Brain function activation map.

Study	Active brain area							
	Frontal lobe	Temporal lobe	Parietal lobe	Occipital lobe	Cerebellum	Basal ganglia	Insula	Pons
Li 2022 [40]	√							
Zhao 2017 [32]	√		√		√			
Li 2015 [28]	√	√	√	√			√	
Gu 2013 [27]		√	√	√		√		
Cai 2012 [25]	√		√					
Wu 2017 [29]	√	√	√					
Liu 2021 [39]	√	√	√					
Liu 2020 [35]			√	√	√			
Zhang 2012 [26]	√	√	√	√	√	√	√	√
Ye 2018 [33]	√		√				√	
Zhang 2020 [38]	√		√					
Gu 2020 [36]	√		√					
Li 2020 [37]	√		√					
Zhang 2019 [34]	√							
Shen 2017 [31]	√		√					
Gong 2017 [30]	√		√		√			
Xing 2013 [41]	√							
Total	15	5	14	4	4	2	3	1

### 3.3 Quality assessment

**Fig 2:**
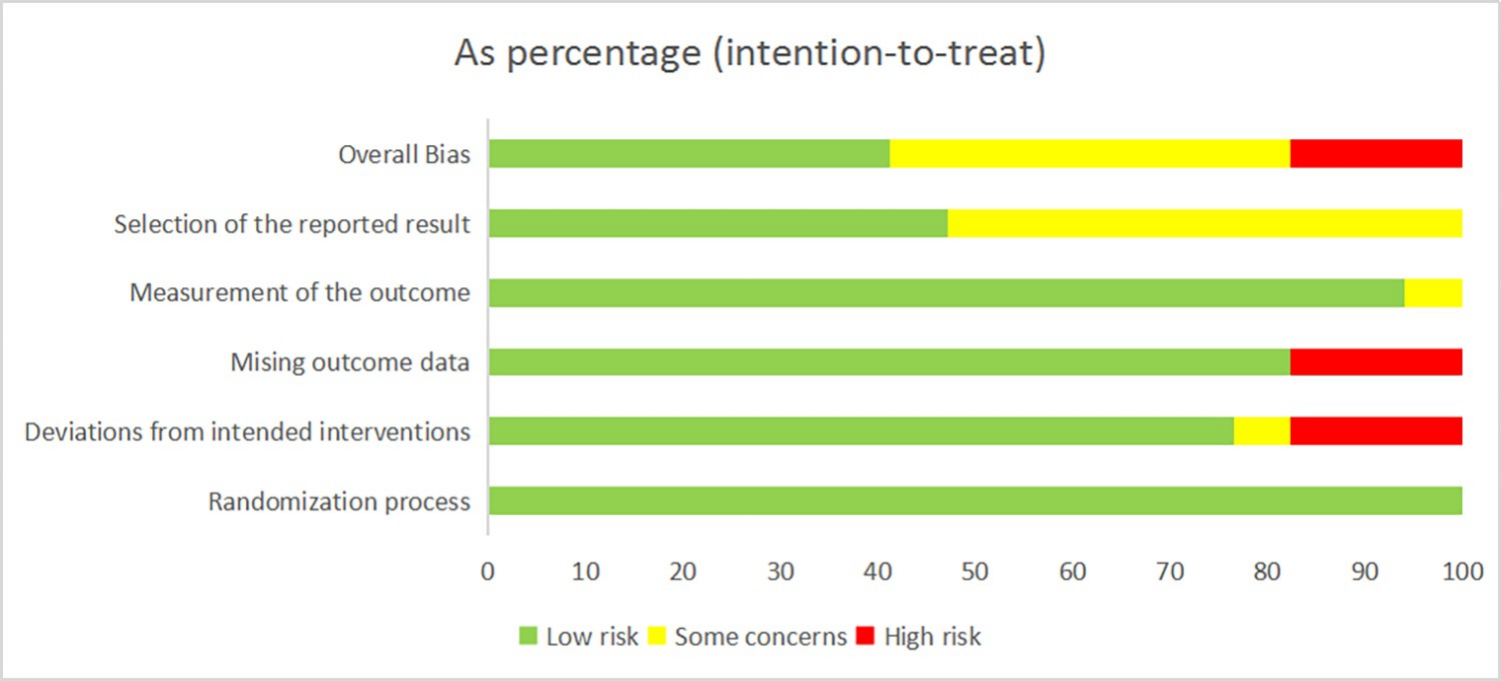
Assessment of risk of bias summary of included studies using the Cochrane tool.

**Fig 3:**
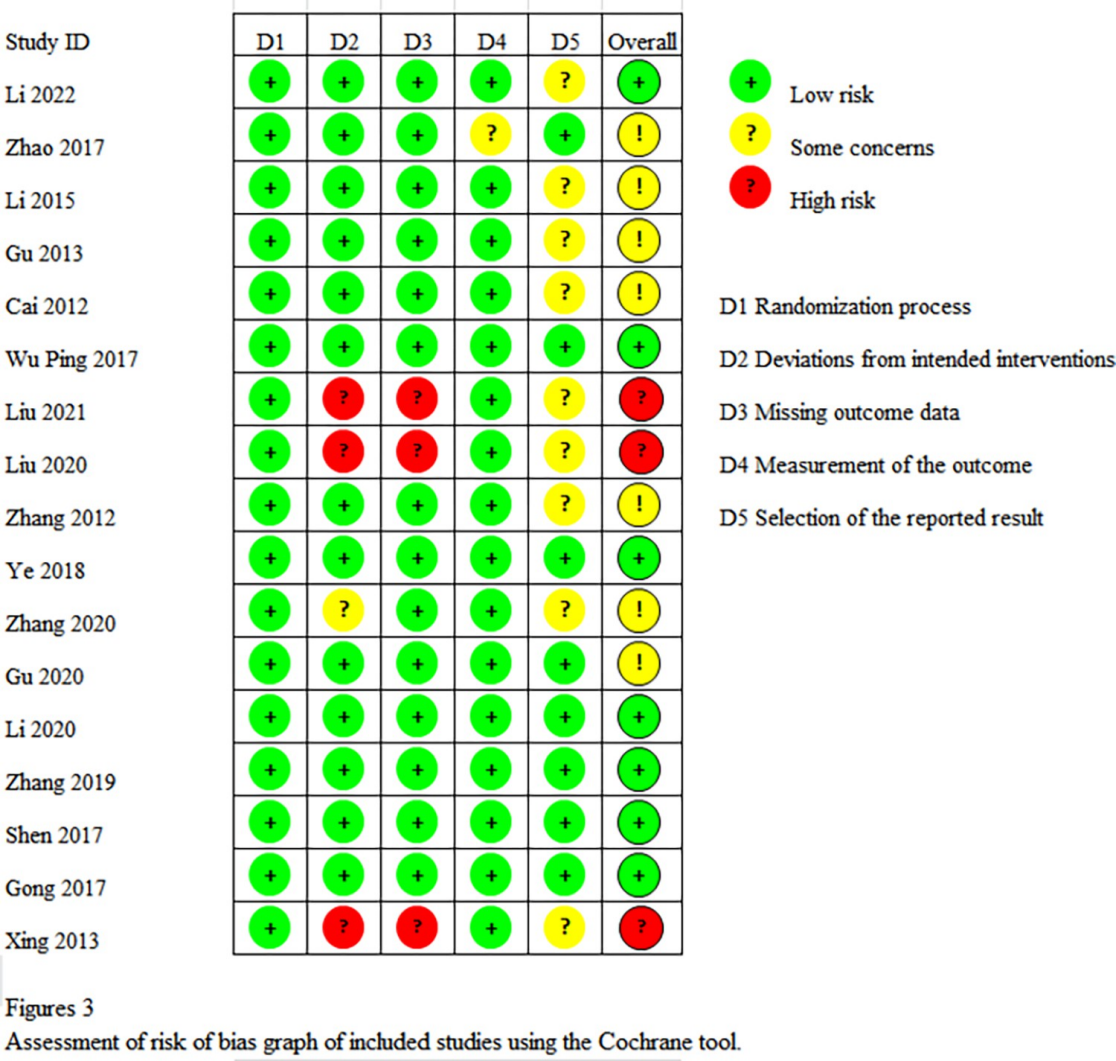
Assessment of risk of bias graph of included studies using the Cochrane tool.

The quality of the 17 included RCTs was generally “low to moderate”. It is important to note that none of the studies mentioned the use of patient blinding and blinding of outcome assessment. Seven studies (n = 7) were considered to be at risk of “some concern”, 7 studies were at low risk, and 3 studies were at high risk of bias. Regarding the randomization process, most studies were considered low risk because the randomization process was reported in detail and the most commonly used technique was the random number table method. The specific quality assessment is shown in Figs 2 and 3.

### 3.4 Meta-analysis

#### 3.4.1 Data analysis of brain function activation area SM1

**Fig 4:**
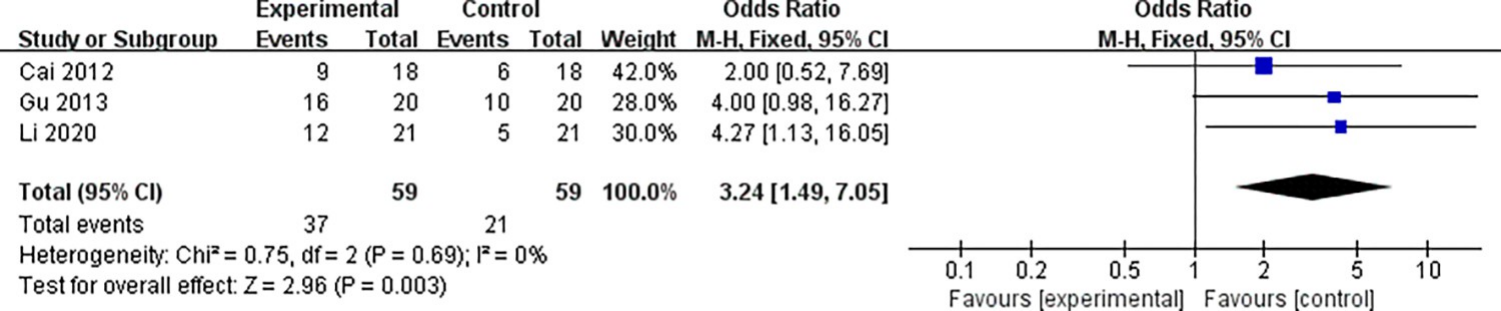
Forest plot of SM1 activation in the brain functional area of the two groups after treatment.

Given that the specific number of people in the same brain function activation zone was not accurately reported in the multiple included studies, from the included studies, a total of 3 studies [25,27,37,42,43] containing the specific effective number of activated brain function areas were selected for further analysis, select brain function SM1 area as the target area for research and analysis. The 3 literatures in this study have been tested for heterogeneity, it suggests that there is no heterogeneity in the literature selected for this study (*I*^2^ = 0% < 50%, *P* = 0.69 > 0.1), The results suggest that compared with basic treatment alone, acupuncture and rehabilitation therapy have a more significant effect on the activated SM1 area of brain function after ischemic stroke (OR = 3.24; 95%CI: 1.49~7.05, *Z* = 2.96, *P* = 0.003 < 0.05), See the Fig 4 below for details.

#### 3.4.2 Data analysis of NIHSS scores

**Fig 5:**
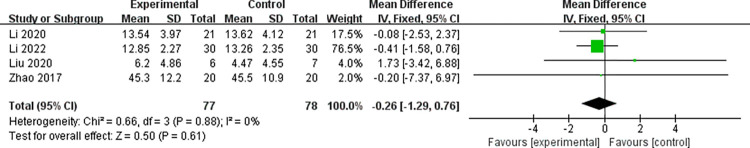
The forest plot of NIHSS in the two groups at baseline.

**Fig 6:**
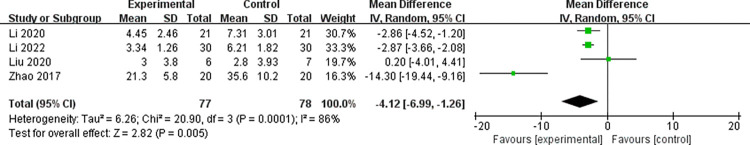
Forest plot of NIHSS scores after treatment in the two groups.

For the 4 studies [32,35,37,40,42] that conducted data analysis on NIHSS Scores. From the above forest plot, it can be clearly seen that there is no heterogeneity in the baseline difference (effect size) of the NIHSS scores between the two groups (*I*^2^ = 0% < 50%, *P* = 0.88 > 0.1). That is, there was no difference in NIHSS scores between the two groups at baseline (MD = -0.26; 95%CI: -1.29~0.67, Z = 0.50, *P* = 0.61 > 0.05), and subsequent meta-analysis can be performed. The consistency results of the baseline period are shown in Fig 5. For the 4 literature in this study, NIHSS scores after treatment tested for heterogeneity, suggesting that there is strong heterogeneity among the documents selected in this study (*I*^2^ = 86% > 50%, *P* = 0.0001), and differences between studies were statistically significant. Subgroup analysis showed that the NIHSS score of the intervention group was significantly lower than that of the control group after treatment, and the intervention effect was significant (MD = -4.12; 95%CI: -6.99~ -1.26, Z = 2.82, *P* = 0.005 < 0.05), see the Fig 6 below for details.

#### 3.4.3 Data analysis of FMA scores

**Fig 7:**
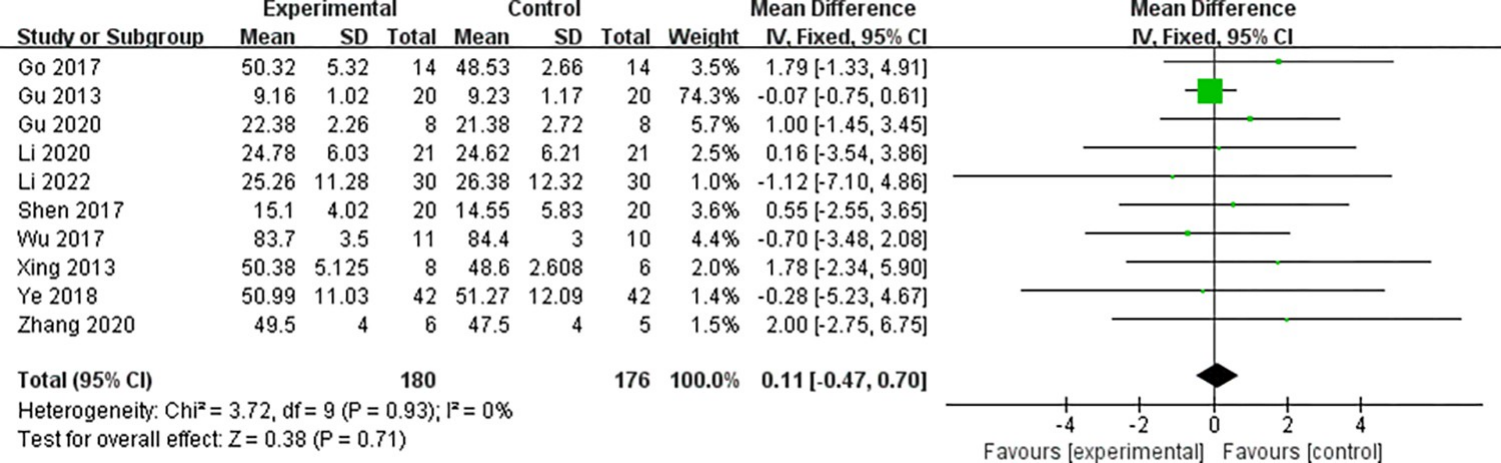
The forest plot of FMA in the two groups at baseline.

**Fig 8:**
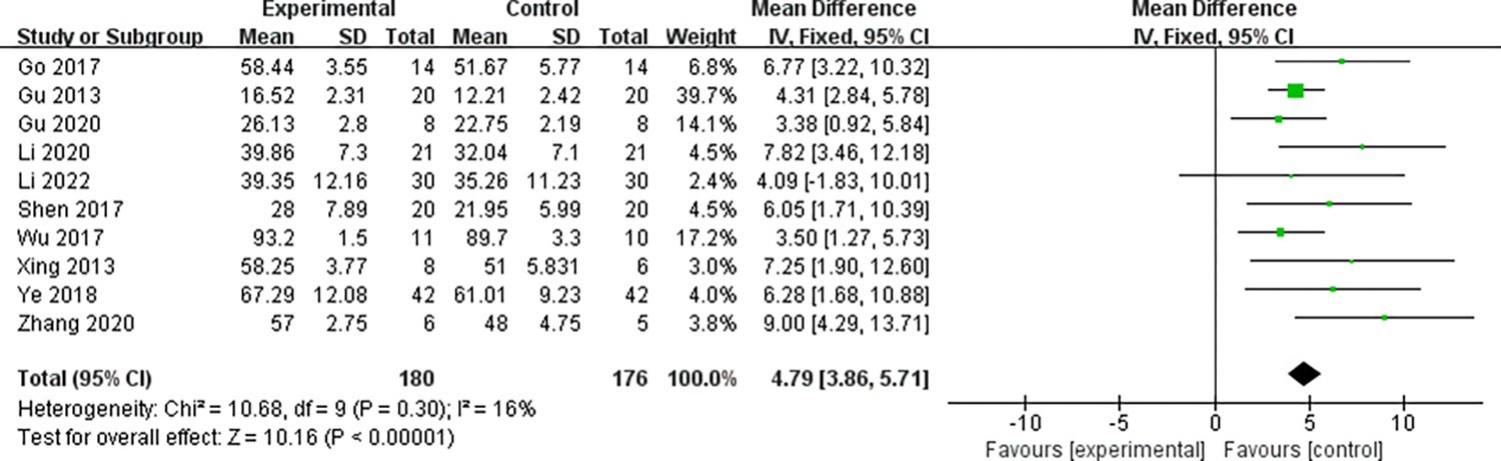
Forest plot of FMA scores after treatment in the two groups.

For the 10 studies [27,29–31,33,36–38,40–42,44] that conducted data analysis on FMA scores. From the above forest plot, it can be clearly seen that there is no heterogeneity in the baseline difference (effect size) of the FMA scores between the two groups (*I*^2^ = 0% < 50%, *P* = 0.93 > 0.1). That is, there was no difference in FMA scores between the two groups at baseline (MD = 0.11; 95%CI: -0.47~0.70, Z = 0.38, *P* = 0.71 > 0.05), and subsequent meta-analysis can be performed. The consistency results of the baseline period are shown in Fig 7. For the 10 literature in this study, FMA scores after treatment tested for heterogeneity, Indicating the absence of heterogeneity in the literature selected for this study (*I*^2^ = 16% < 50%, *P* = 0.30). Subgroup analysis showed that the FMA score of the intervention group was significantly higher than that of the control group after treatment, and the intervention effect was significant (MD = 4.79; 95%CI: 3.86~ 5.71, Z = 10.16, *P* < 0.00001), see the Fig 8 below for details.

#### 3.4.4 Evaluation of publication bias

**Fig 9:**
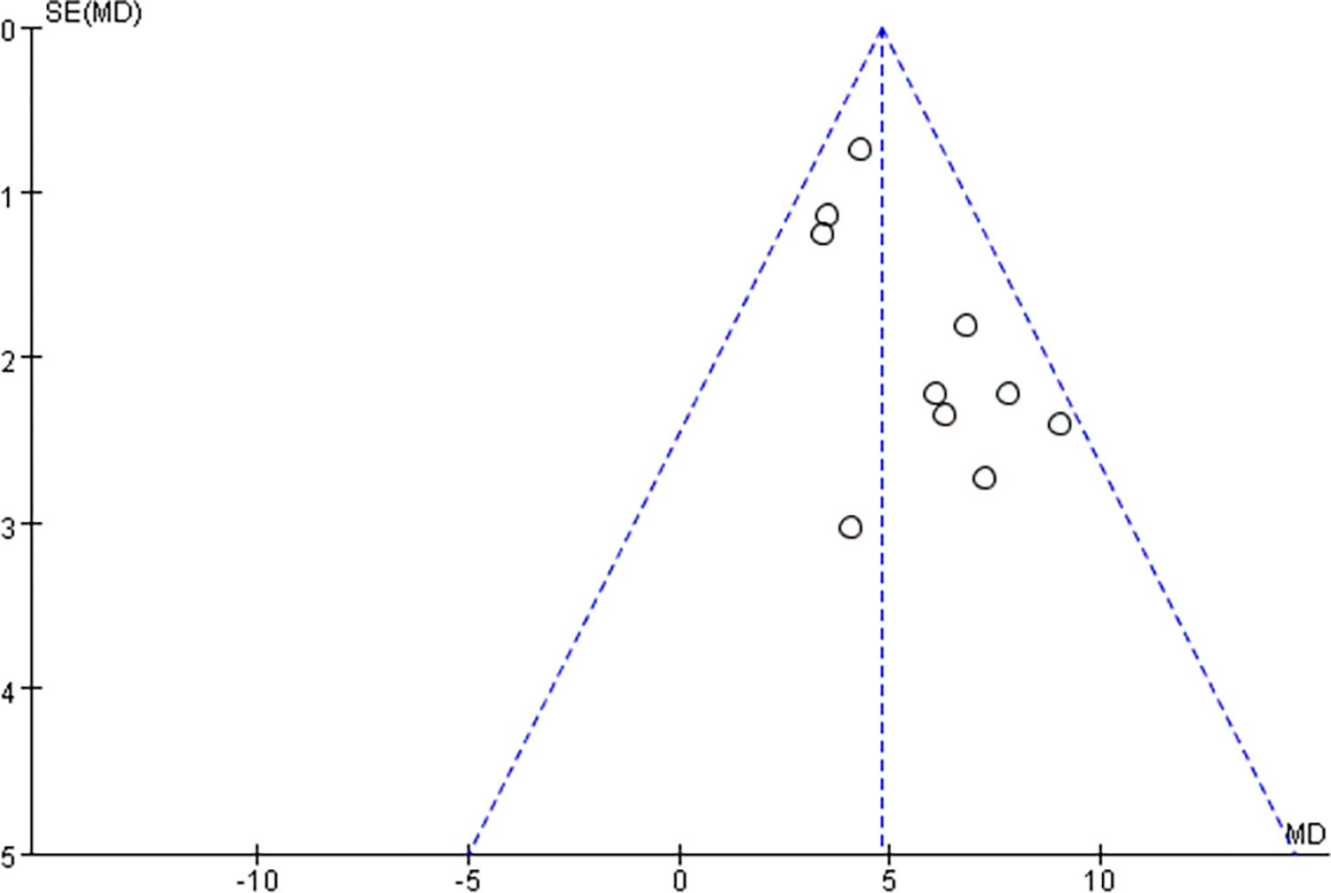
Publication bias of clinical efficacy.

By drawing a funnel diagram to investigate whether there is publication bias in the 10 literatures in this study, it is found that the distribution of each point is symmetrical through visual inspection, and the possibility of publication bias is very small. The conclusion that the funnel diagram is symmetrical and there is no publication bias is concluded, which suggests the significance of this study. The conclusion is accurate and reliable. See Fig 9 below for details.

## 4. Discussion

Traditional Chinese medicine believes that stroke is mostly related to the heart, liver, spleen, kidneys, and internal organs. Due to the weakness of righteousness, the internal movement of liver wind, and the invasion of external evils, blood qi is blocked, and the meridian qi and blood are lost for nourishment, ascending to the brain, closing the clear orifices and seeing sudden fainting, hemiplegia and other symptoms [45]. Acupuncture can dredge qi and blood, adjust the excess and decline of yin and yang, dredge the meridians, reconcile qi and blood, and then promote the improvement of the brain and the organs of the body. Studies have reported that acupuncture on bilateral motor and sensory areas can improve the motor function of stroke patients with hemiplegia, promote the balance of muscle tension, relieve spasticity, and reduce various indicators of blood rheology [46]. Wu et al [47] found through research that acupuncture at acupoints can cause changes in the BOLD signal in the relevant areas of the brain indicating that the local cerebral blood flow has changed that is, the local brain function has changed. It has been reported that acupuncture GB34 can enhance the FC decline between bilateral M1 in right hemisphere subcortical stroke patients, which may reflect the neural plasticity mechanism of acupuncture treatment of stroke in the brain center [48]. Acupuncture can improve the clinical manifestations of ischemic stroke, promote recovery from the disease, rebuild neuroplasticity of brain function under the mechanism of damaged brain function, and enhance the recovery of neurological function. Stroke is one of the dominant diseases treated with acupuncture. Acupuncture and rehabilitation, as a pure natural therapy, not only has good curative effect, but also has the unique advantage of no toxic and side effects. In the treatment, it can play a role through multiple links and multiple targets, thereby stimulating the overall regulation function of the body [49].

This meta-analysis included 17 randomized controlled studies, including 699 patients, with a maximum sample size of 144 cases and a minimum sample size of 11 cases. Through the collation and analysis of the literature, in the case of a large sample size, it was found that acupuncture and rehabilitation treatment of ischemic stroke can significantly improve the curative effect of patients, which is consistent with the conclusions reported by most studies. Studies have reported that electroacupuncture stimulation of sensory areas of the scalp combined with sensory retraining can help improve synaptic efficiency, accelerate nerve function reorganization, and promote disease recovery [27]. Acupuncture can more extensively activate relevant brain functional areas in patients with ischemic stroke, and it may also be one of the important mechanisms for brain function recovery and neurological remodeling [25]. This meta-analysis also evaluated the efficacy of acupuncture and rehabilitation in treating ischemic stroke in terms of motor function and activities of daily living. The results of the FMA assessment scale of limb motor function showed that acupuncture can improve the motor function of the limbs and strengthen the coordination function of the limbs. The NIHSS scale reflects the impairment of neurological function, indicating that acupuncture can improve the impairment of neurological function and promote the recovery of neurological function. Therefore, this meta-analysis re-collects and organizes multiple research data, and further conducts integrated analysis to study the treatment of ischemic stroke with acupuncture and rehabilitation. Previous studies have repeatedly proved the effectiveness of acupuncture and rehabilitation for ischemic stroke, and can improve the limb dysfunction of patients. Starting from the SM1 brain area, the effectiveness and reliability of the treatment methods are proved by supplementary neurological function assessment tables and limb dysfunction scores. In terms of activation of functional areas of the brain, acupuncture and rehabilitation therapy are more effective than basic therapy, and the curative effect is also more significant. Regarding the improvement of neurological deficits, compared with basic treatment of Western medicine, acupuncture and rehabilitation treatment have more significant effects.

It was observed in this study that the frontal and parietal lobes were most activated during acupuncture and rehabilitation. The frontal lobe is located in the front part of the cerebral cortex and is involved in the regulation of movement, cognition, emotion, language and other functions. The parietal lobe controls sensory and cognitive functions. Both encompass motor and cognitive functions. In terms of movement, it has the motor ability to control voluntary movements of the contralateral half of the body, control coordinated movements, and also control eye movements and fine movements of the hands. The cognitive aspect includes normal functions such as memory, judgment, abstraction, and thinking. After stroke, neurological dysfunction often occurs. It has been confirmed that acupuncture can effectively improve neurological dysfunction after stroke, but the specific internal regulatory mechanisms have not been studied in depth. In this study, it was found that the degree of activation of the frontal and parietal lobes under acupuncture rehabilitation reflects the interventional stimulation of neurological function after acupuncture rehabilitation for stroke. Does it indicate the creation of targeted treatments for neurological deficits? Central regulation is carried out from the brain areas that control motor and cognitive functions, thereby improving limb dysfunction and cognitive dysfunction after stroke.

However, from a comprehensive analysis, most clinical studies do not have a unified quantitative standard for the changes in brain functional areas in the treatment of ischemic stroke, and the directions are diversified. There is a lack of in-depth research on a single brain area, and no in-depth mechanism exploration has been carried out. The mechanism of activation of specific brain regions has not been clarified, and research on targeted changes remains to be explored. Especially in terms of the activation of specific therapeutic effects on specific brain regions, finding the best activation method for this brain region can help to quickly carry out targeted treatment in the clinic, thereby speeding up the improvement of the patient’s disease condition and shortening the treatment cycle. Combined with the area of disease damage, choosing the treatment method with the best activation rate can bring better clinical outcomes, reduce the pain of patients and certain economic pressure, and reduce the occurrence of various stroke sequelae.

## 5. Conclusion

This meta-analysis indicated that acupuncture rehabilitation has clinical efficacy in the treatment of ischemic stroke by activating the corresponding brain functional areas, improving motor dysfunction and neurological deficit. The studies included in this meta-analysis were all randomized controlled trials, and the outcome has high scientific evidence. However, only a few of the included studies specifically mentioned the corresponding brain functional activation areas, and most of them only mentioned the degree of functional connectivity and the responses of various parts of the brain to acupuncture. At the same time, through the quality evaluation, it can be known that the included studies have high risk evidence in the blind design, and the included studies rarely report the effectiveness of the treatment, making the effectiveness of the specific treatment effect uncertain. In addition, most of the experiments are from Chinese databases, and a few are from SCI databases. Their language and region may also lead to bias, and there is a certain regional applicability.

At the same time, through this meta-analysis, it was found that different acupoint groups and acupuncture methods were different, resulting in different brain tissue activities and brain functional regulation. We hope that future clinical research should adopt high-quality randomized double-blind controlled trials, carry out more detailed and larger sample design, long-term efficacy evaluation, and evidence-based research methodology. Moreover, acupuncture procedures should be more standardized and unified. This can reduce the one-sidedness, heterogeneity and chance of research reports, and make the research evidence more scientific.

## Supporting information

S1 Appendix The PRISMA checklist. (DOCX)

S2 Appendix Search strategy. (DOCX)
