# Supplementary material for: Efficacy of acupuncture and rehabilitation therapy on brain function activation area and neurological function in ischemic stroke: A systematic review and meta-analysis
Source: PLoS One. 2024 Feb 23;19(2):e0298547. doi: 10.1371/journal.pone.0298547 (PMC10889652; doi:10.1371/journal.pone.0298547)
Supplement: S2 Appendix — (DOCX) [file pone.0298547.s002.docx]

Supplementary Material

**Efficacy of acupuncture and rehabilitation therapy on brain function activation area and neurological function in ischemic stroke: a systematic review and meta-analysis**

**Tao Zhu^1,2¶^, Yihao Zhou^3¶^, Anhong Dai^4^, Song Li^1^, Li Zhou^1^, Xiahui Zhang^2^ , Wei Zhang^4^, Jing Shi^1,2*^**

^1^ Yunnan University of Chinese Medicine, Kunming, China. ^2^ The First Affiliated Hospital of Yunnan University of Chinese Medicine, Yunnan Provincial Hospital of Traditional Chinese Medicine, Kunming, China. ^3^ Heilongjiang University of Chinese Medicine, Harbin, China. ^4^ Yan' an Hospital Affiliated to Kunming Medical University, Kunming, China.

*** Correspondence:** (E-mail: 2662831291@qq.com)

**Supplementary Materials**

**Search strategy**

| PUBMED | |
| --- | --- |
| Acupuncture search strategy | (((("Ischemic Stroke"[Mesh]) OR ((((((((((((((((((((((((Ischemic Strokes[Title/Abstract]) OR (Stroke, Ischemic[Title/Abstract])) OR (Ischaemic Stroke[Title/Abstract])) OR (Ischaemic Strokes[Title/Abstract])) OR (Stroke, Ischaemic[Title/Abstract])) OR (Cryptogenic Ischemic Stroke[Title/Abstract])) OR (Cryptogenic Ischemic Strokes[Title/Abstract])) OR (Ischemic Stroke, Cryptogenic[Title/Abstract])) OR (Stroke, Cryptogenic Ischemic[Title/Abstract])) OR (Cryptogenic Stroke[Title/Abstract])) OR (Cryptogenic Strokes[Title/Abstract])) OR (Stroke, Cryptogenic[Title/Abstract])) OR (Cryptogenic Embolism Stroke[Title/Abstract])) OR (Cryptogenic Embolism Strokes[Title/Abstract])) OR (Embolism Stroke, Cryptogenic[Title/Abstract])) OR (Stroke, Cryptogenic Embolism[Title/Abstract])) OR (Wake-up Stroke[Title/Abstract])) OR (Stroke, Wake-up[Title/Abstract])) OR (Wake up Stroke[Title/Abstract])) OR (Wake-up Strokes[Title/Abstract])) OR (Acute Ischemic Stroke[Title/Abstract])) OR (Acute Ischemic Strokes[Title/Abstract])) OR (Ischemic Stroke, Acute[Title/Abstract])) OR (Stroke, Acute Ischemic[Title/Abstract]))) AND ((((((((acupuncture[Title/Abstract]) OR (acupuncture therapy[Title/Abstract])) OR (acupuncture treatment[Title/Abstract])) OR (electroacupuncture[Title/Abstract])) OR (ear acupuncture[Title/Abstract])) OR (acupuncture, ear[Title/Abstract])) OR (auriculotherapy[Title/Abstract])) OR (scalp acupuncture[Title/Abstract]))) AND (((((fMRI[Title/Abstract]) OR (MRI[Title/Abstract])) OR (functional magnetic resonance[Title/Abstract])) OR (Resting state magnetic resonance[Title/Abstract])) OR (brain function[Title/Abstract]))) AND ((randomized controlled trial[Publication Type] OR randomized[Title/Abstract] OR placebo[Title/Abstract])) |
| Rehabilitation retrieval strategy | ((((ischemic stroke) OR ((((((((((((((((((((((((ischemic stroke[Title/Abstract]) OR (Stroke, Ischemic[Title/Abstract])) OR (Ischaemic Stroke[Title/Abstract])) OR (Ischaemic Strokes[Title/Abstract])) OR (Stroke, Ischaemic[Title/Abstract])) OR (Cryptogenic Ischemic Stroke[Title/Abstract])) OR (Cryptogenic Ischemic Strokes[Title/Abstract])) OR (Ischemic Stroke, Cryptogenic[Title/Abstract])) OR (Stroke, Cryptogenic Ischemic[Title/Abstract])) OR (Cryptogenic Stroke[Title/Abstract])) OR (Cryptogenic Strokes[Title/Abstract])) OR (Stroke, Cryptogenic[Title/Abstract])) OR (Cryptogenic Embolism Stroke[Title/Abstract])) OR (Cryptogenic Embolism Strokes[Title/Abstract])) OR (Embolism Stroke, Cryptogenic[Title/Abstract])) OR (Stroke, Cryptogenic Embolism[Title/Abstract])) OR (Wake-up Stroke[Title/Abstract])) OR (Stroke, Wake-up[Title/Abstract])) OR (Wake up Stroke[Title/Abstract])) OR (Wake-up Strokes[Title/Abstract])) OR (Acute Ischemic Stroke[Title/Abstract])) OR (Acute Ischemic Strokes[Title/Abstract])) OR (Ischemic Stroke, Acute[Title/Abstract])) OR (Stroke, Acute Ischemic[Title/Abstract]))) AND ((((((occupational therapy[Title/Abstract]) OR (physical therapy[Title/Abstract])) OR (Exercise therapy[Title/Abstract])) OR (Speech therapy[Title/Abstract])) OR (rehabilitation training[Title/Abstract])) OR (rehabilitation[Title/Abstract]))) AND ((((((fMRI[Title/Abstract]) OR (MRI[Title/Abstract])) OR (functional magnetic resonance[Title/Abstract])) OR (Resting state magnetic resonance[Title/Abstract])) OR (brain function[Title/Abstract]))))) AND (((randomized controlled trial[Publication Type] OR randomized[Title/Abstract] OR placebo[Title/Abstract]))) |
| Embase | |
| Rehabilitation retrieval strategy | #41. #26 AND #33 AND #39 AND #40  #40. 'random':ab,ti OR 'placebo':ab,ti OR 'double-blind':ab,ti  #39. #34 OR #35 OR #36 OR #37 OR #38  #38. 'brain function':ab,ti  #37. 'resting state magnetic resonance':ab,ti  #36. 'functional magnetic resonance':ab,ti  #35. 'mri':ab,ti  #34. 'fmri':ab,ti  #33. #27 OR #28 OR #29 OR #30 OR #31 OR #32  #32. 'rehabilitation':ab,ti  #31. 'rehabilitation training':ab,ti  #30. 'speech therapy':ab,ti  #29. 'exercise therapy':ab,ti  #28. 'physical therapy':ab,ti  #27. 'occupational therapy':ab,ti  #26. #1 OR #2 OR #3 OR #4 OR #5 OR #6 OR #7 OR #8 OR #9 OR #10 OR #11 OR #12 OR #13 OR #14 OR #15 OR #16 OR #17 OR #18 OR #19 OR #20 OR #21 OR #22 OR #23 OR #24 OR #25  #25. 'stroke, acute ischemic':ti,ab  #24. 'ischemic stroke, acute':ti,ab  #23. 'acute ischemic strokes':ti,ab  #22. 'acute ischemic stroke':ti,ab  #21. 'wake-up strokes':ti,ab  #20. 'wake up stroke':ti,ab  #19. 'stroke, wake-up':ti,ab  #18. 'wake-up stroke':ti,ab  #17. 'stroke, cryptogenic embolism':ti,ab  #16. 'embolism stroke, cryptogenic':ti,ab  #15. 'cryptogenic embolism strokes':ti,ab  #14. 'cryptogenic embolism stroke':ti,ab  #13. 'stroke, cryptogenic':ti,ab  #12. 'cryptogenic strokes':ti,ab  #11. 'cerebrovascular accident':ti,ab  #10. 'stroke, cryptogenic ischemic':ti,ab  #9. 'ischemic stroke, cryptogenic':ti,ab  #8. 'cryptogenic ischemic strokes':ti,ab  #7. 'cryptogenic ischemic stroke':ti,ab  #6. 'stroke, ischaemic':ti,ab  #5. 'ischaemic strokes':ti,ab  #4. 'ischemic stroke':ti,ab  #3. 'stroke, ischemic':ti,ab  #2. 'ischemic strokes':ti,ab  #1. ischemic AND ('stroke'/exp OR stroke) |
| Acupuncture search strategy | #43. #26 AND #35 AND #41 AND #42  #42. #36 OR #37 OR #38 OR #39 OR #40  #41. 'random':ab,ti OR 'placebo':ab,ti OR 'double-blind':ab,ti  #40. 'brain function':ab,ti  #39. 'resting state magnetic resonance':ab,ti  #38. 'functional magnetic resonance':ab,ti  #37. 'mri':ab,ti  #36. 'fmri':ab,ti  #35. #27 OR #28 OR #29 OR #30 OR #31 OR #32 OR #33 OR  #34. 'scalp acupuncture':ab,ti  #33. 'auriculotherapy':ab,ti  #32. 'acupuncture, ear':ab,ti  #31. 'ear acupuncture':ab,ti  #30. 'electroacupuncture':ab,ti  #29. 'acupuncture treatment':ab,ti  #28. 'acupuncture therapy':ab,ti  #27. 'acupuncture':ab,ti  #26. #1 OR #2 OR #3 OR #4 OR #5 OR #6 OR #7 OR #8 OR #9 OR #10 OR #11 OR #12 OR #13 OR #14 OR #15 OR #16 OR #17 OR #18 OR #19 OR #20 OR #21 OR #22 OR #23 OR #24 OR #25  #25. 'stroke, acute ischemic':ab,ti  #24. 'ischemic stroke, acute':ab,ti  #23. 'acute ischemic strokes':ab,ti  #22. 'acute ischemic stroke':ab,ti  #21. 'wake-up strokes':ab,ti  #20. 'wake up stroke':ab,ti  #19. 'stroke, wake-up':ab,ti  #18. 'wake up stroke':ab,ti  #17. 'stroke, cryptogenic embolism':ab,ti  #16. 'embolism stroke, cryptogenic':ab,ti  #15. 'cryptogenic embolism strokes':ab,ti  #14. 'cryptogenic embolism stroke':ab,ti  #13. 'stroke, cryptogenic':ab,ti  #12. 'cryptogenic strokes':ab,ti  #11. 'cryptogenic stroke':ab,ti  #10. 'stroke, cryptogenic ischemic':ab,ti  #9. 'ischemic stroke, cryptogenic':ab,ti  #8. 'cryptogenic ischemic strokes':ab,ti  #7. 'cryptogenic ischemic stroke':ab,ti  #6. 'stroke, ischaemic':ab,ti  #5. 'ischaemic strokes':ab,ti  #4. 'ischaemic stroke':ab,ti  #3. 'stroke, ischemic':ab,ti  #2. 'ischemic strokes':ab,ti  #1. ischemic AND ('stroke'/exp OR stroke) |
| Cochrane library(Rehabilitation retrieval strategy) | |
| #1 | MeSH descriptor: [Ischemic Stroke] explode all trees |
| #2 | (Ischemic Strokes):ti,ab,kw OR (Stroke, Ischemic):ti,ab,kw OR (Ischaemic Stroke):ti,ab,kw OR (Ischaemic Strokes):ti,ab,kw OR (Stroke, Ischaemic):ti,ab,kw (Word variations have been searched) |
| #3 | (Cryptogenic Ischemic Stroke):ti,ab,kw OR (Cryptogenic Ischemic Strokes):ti,ab,kw OR (Ischemic Stroke, Cryptogenic):ti,ab,kw OR (Stroke, Cryptogenic Ischemic):ti,ab,kw OR (Cryptogenic Stroke):ti,ab,kw (Word variations have been searched) |
| #4 | (Cryptogenic Strokes):ti,ab,kw OR (Stroke, Cryptogenic):ti,ab,kw OR (Cryptogenic Embolism Stroke):ti,ab,kw OR (Cryptogenic Embolism Strokes):ti,ab,kw OR (Embolism Stroke, Cryptogenic):ti,ab,kw (Word variations have been searched) |
| #5 | (Stroke, Cryptogenic Embolism):ti,ab,kw OR (Wake-up Stroke):ti,ab,kw OR (Stroke, Wake-up):ti,ab,kw OR (Wake up Stroke):ti,ab,kw OR (Wake-up Strokes):ti,ab,kw (Word variations have been searched) |
| #6 | (Acute Ischemic Stroke):ti,ab,kw OR (Acute Ischemic Strokes):ti,ab,kw OR (Ischemic Stroke, Acute):ti,ab,kw OR (Stroke, Acute Ischemic):ti,ab,kw (Word variations have been searched) |
| #7 | #1 or #2 or #3 or #4 or #5 or #6 |
| #8 | (fMRI):ti,ab,kw OR (MRI):ti,ab,kw OR (functional magnetic resonance):ti,ab,kw AND (Resting state magnetic resonance):ti,ab,kw AND (brain function):ti,ab,kw (Word variations have been searched) |
| #9 | (occupational therapy):ti,ab,kw OR (physical therapy):ti,ab,kw OR (Exercise therapy):ti,ab,kw OR (Speech therapy):ti,ab,kw OR (rehabilitation training):ti,ab,kw (Word variations have been searched) |
| #10 | (rehabilitation):ti,ab,kw (Word variations have been searched) |
| #11 | #9 or #10 |
| #12 | #7 and #8 and #11 |
| Cochrane library(Acupuncture search strategy) | |
| #1 | MeSH descriptor: [Ischemic Stroke] explode all trees |
| #2 | (Ischemic Strokes):ti,ab,kw OR (Stroke, Ischemic):ti,ab,kw OR (Ischaemic Stroke):ti,ab,kw OR (Ischaemic Strokes):ti,ab,kw OR (Stroke, Ischaemic):ti,ab,kw (Word variations have been searched) |
| #3 | (Cryptogenic Ischemic Stroke):ti,ab,kw OR (Cryptogenic Ischemic Strokes):ti,ab,kw OR (Ischemic Stroke, Cryptogenic):ti,ab,kw OR (Stroke, Cryptogenic Ischemic):ti,ab,kw OR (Cryptogenic Stroke):ti,ab,kw (Word variations have been searched) |
| #4 | (Cryptogenic Strokes):ti,ab,kw OR (Stroke, Cryptogenic):ti,ab,kw OR (Cryptogenic Embolism Stroke):ti,ab,kw OR (Cryptogenic Embolism Strokes):ti,ab,kw OR (Embolism Stroke, Cryptogenic):ti,ab,kw (Word variations have been searched) |
| #5 | (Stroke, Cryptogenic Embolism):ti,ab,kw OR (Wake-up Stroke):ti,ab,kw OR (Stroke, Wake-up):ti,ab,kw OR (Wake up Stroke):ti,ab,kw OR (Wake-up Strokes):ti,ab,kw (Word variations have been searched) |
| #6 | (Acute Ischemic Stroke):ti,ab,kw OR (Acute Ischemic Strokes):ti,ab,kw OR (Ischemic Stroke, Acute):ti,ab,kw OR (Stroke, Acute Ischemic):ti,ab,kw (Word variations have been searched) |
| #7 | #1 or #2 or #3 or #4 or #5 or #6 |
| #8 | (acupuncture):ti,ab,kw OR (acupuncture therapy):ti,ab,kw OR (acupuncture treatment):ti,ab,kw OR (electroacupuncture):ti,ab,kw AND (ear acupuncture):ti,ab,kw (Word variations have been searched) |
| #9 | (acupuncture, ear):ti,ab,kw OR (auriculotherapy):ti,ab,kw OR (scalp acupuncture):ti,ab,kw (Word variations have been searched) |
| #10 | #8 or #9 |
| #11 | (fMRI):ti,ab,kw OR (MRI):ti,ab,kw OR (functional magnetic resonance):ti,ab,kw AND (Resting state magnetic resonance):ti,ab,kw AND (brain function):ti,ab,kw (Word variations have been searched) |
| #12 | #7 and #10 and #11 |
| China National Knowledge Infrastructure (CNKI) | |
| SU=（'脑梗死'+'脑梗塞'+'中风'+'缺血性脑卒中'+'脑血管意外'） AND SU=（'作业疗法'+'物理疗法'+'运动疗法'+'语言疗法'+'康复训练'+'康复'） AND SU=（'fMRI'+'MRI'+'功能性磁共振'+'静息态磁共振'+'脑功能'） AND AB = ('随机对照实验'+'随机对照试验'+'随机对照研究'+'随机对照'+'RCT'+'随机') | |
| SU=（'脑梗死'+'脑梗塞'+'中风'+'缺血性脑卒中'+'脑血管意外'） AND SU=（'针灸'+'针刺'+'体针'+'头针'+'电针'+'温针灸'+'针'） AND SU=（'fMRI'+'MRI'+'功能性磁共振'+'静息态磁共振'+'脑功能'） AND AB = ('随机对照实验'+'随机对照试验'+'随机对照研究'+'随机对照'+'RCT'+'随机') | |
| Wangfang Database | |
| (主题:( "脑梗死" OR "脑梗塞" OR "中风" OR "缺血性脑卒中" OR "脑血管意外") and 主题:("作业疗法" OR "物理疗法"OR "运动疗法" OR "语言疗法" OR "康复训练" OR "康复") and 主题:("fMRI" OR "MRI" OR "功能性磁共振" OR "静息态磁共振" OR "脑功能") and 主题:("随机对照实验"OR"随机对照试验"OR"随机对照研究"OR"随机对照"OR"RCT"OR"随机")) and Date:2000-2023 | |
| (主题:( "脑梗死" OR "脑梗塞" OR "中风" OR "缺血性脑卒中" OR "脑血管意外") and 主题:("针灸" OR "针刺"OR "体针" OR "头针" OR "电针" OR "温针灸"OR "针") and 主题:("fMRI" OR "MRI" OR "功能性磁共振" OR "静息态磁共振" OR "脑功能") and 主题:("随机对照实验"OR"随机对照试验"OR"随机对照研究"OR"随机对照"OR"RCT"OR"随机")) and Date:2000-2023 | |
| Chinese BioMedical Literature Database | |
| (((("随机对照实验"[常用字段:智能] OR "随机对照研究"[常用字段:智能] OR "随机对照"[常用字段:智能] OR "RCT"[常用字段:智能] OR "随机"[常用字段:智能]) AND 2000-2023[日期]) OR ("随机对照试验"[不加权:扩展])) AND 2000-2023[日期]) AND (("fMRI"[常用字段:智能] OR "MRI"[常用字段:智能] OR "功能性磁共振"[常用字段:智能] OR "静息态磁共振"[常用字段:智能] OR "脑功能"[常用字段:智能]) AND 2000-2023[日期]) AND (("作业疗法"[常用字段:智能] OR "物理疗法"[常用字段:智能] OR "运动疗法"[常用字段:智能] OR "语言疗法"[常用字段:智能] OR "康复训练"[常用字段:智能] OR "康复"[常用字段:智能]) AND 2000-2023[日期]) AND (((("脑梗塞"[常用字段:智能] OR "中风"[常用字段:智能] OR "缺血性脑卒中"[常用字段:智能] OR "脑血管意外"[常用字段:智能]) AND 2000-2023[日期]) OR ("脑梗死"[不加权:扩展])) AND 2000-2023[日期]) | |
| (((("随机对照实验"[常用字段:智能] OR "随机对照研究"[常用字段:智能] OR "随机对照"[常用字段:智能] OR "RCT"[常用字段:智能] OR "随机"[常用字段:智能]) AND 2000-2023[日期]) OR ("随机对照试验"[不加权:扩展])) AND 2000-2023[日期]) AND (("fMRI"[常用字段:智能] OR "MRI"[常用字段:智能] OR "功能性磁共振"[常用字段:智能] OR "静息态磁共振"[常用字段:智能] OR "脑功能"[常用字段:智能]) AND 2000-2023[日期]) AND (("针灸"[常用字段:智能] OR "针刺"[常用字段:智能] OR "体针"[常用字段:智能] OR "头针"[常用字段:智能] OR "电针"[常用字段:智能] OR "温针灸"[常用字段:智能]) AND 2000-2023[日期]) AND (((("脑梗塞"[常用字段:智能] OR "中风"[常用字段:智能] OR "缺血性脑卒中"[常用字段:智能] OR "脑血管意外"[常用字段:智能]) AND 2000-2023[日期]) OR ("脑梗死"[不加权:扩展])) AND 2000-2023[日期]) | |
| Chinese Science and Technology Periodical (VIP) Database | |
| (M=("脑梗死"+"脑梗塞"+"中风"+"缺血性脑卒中"+"脑血管意外") AND ("作业疗法"+"物理疗法"+"运动疗法"+"语言疗法"+"康复训练"+"康复") AND ("fMRI"+"MR"+"功能性磁共振"+"静息态磁共振"+"脑功能")) AND R=("随机对照实验"+"随机对照试验"+"随机对照研究"+"随机对照"+"RCT"+"随机") | |
| (M=("脑梗死"+"脑梗塞"+"中风"+"缺血性脑卒中"+"脑血管意外") AND ("针灸"+"针刺"+"体针"+"头针"+"电针"+"温针灸"+"针") AND ("fMRI"+"MR"+"功能性磁共振"+"静息态磁共振"+"脑功能")) AND R=("随机对照实验"+"随机对照试验"+"随机对照研究"+"随机对照"+"RCT"+"随机") | |
